# Disruption of *Escherichia coli* Nissle 1917 K5 Capsule Biosynthesis, through Loss of Distinct *kfi* genes, Modulates Interaction with Intestinal Epithelial Cells and Impact on Cell Health

**DOI:** 10.1371/journal.pone.0120430

**Published:** 2015-03-19

**Authors:** Jonathan Nzakizwanayo, Sandeep Kumar, Lesley A. Ogilvie, Bhavik A. Patel, Cinzia Dedi, Wendy M. Macfarlane, Brian V. Jones

**Affiliations:** 1 School of Pharmacy and Biomolecular Sciences, University of Brighton, Brighton, East Sussex, United Kingdom; 2 Queen Victoria Hospital NHS Foundation Trust, East Grinstead, West Sussex, United Kingdom; Charité, Campus Benjamin Franklin, GERMANY

## Abstract

*Escherichia coli* Nissle 1917 (EcN) is among the best characterised probiotics, with a proven clinical impact in a range of conditions. Despite this, the mechanisms underlying these "probiotic effects" are not clearly defined. Here we applied random transposon mutagenesis to identify genes relevant to the interaction of EcN with intestinal epithelial cells. This demonstrated mutants disrupted in the *kfiB* gene, of the K5 capsule biosynthesis cluster, to be significantly enhanced in attachment to Caco-2 cells. However, this phenotype was distinct from that previously reported for EcN K5 deficient mutants (*kfiC* null mutants), prompting us to explore further the role of *kfiB* in EcN:Caco-2 interaction. Isogenic mutants with deletions in *kfiB* (EcNΔ*kfiB*), or the more extensively characterised K5 capsule biosynthesis gene *kfiC* (EcNΔ*kfiC*), were both shown to be capsule deficient, but displayed divergent phenotypes with regard to impact on Caco-2 cells. Compared with EcNΔ*kfiC* and the EcN wild-type, EcNΔ*kfiB* exhibited significantly greater attachment to Caco-2 cells, as well as apoptotic and cytotoxic effects. In contrast, EcNΔ*kfiC* was comparable to the wild-type in these assays, but was shown to induce significantly greater COX-2 expression in Caco-2 cells. Distinct differences were also apparent in the pervading cell morphology and cellular aggregation between mutants. Overall, these observations reinforce the importance of the EcN K5 capsule in host-EcN interactions, but demonstrate that loss of distinct genes in the K5 pathway can modulate the impact of EcN on epithelial cell health.

## Introduction

Due to the intimate role of the gut microbiome in human health and disease processes, this predominantly bacterial community is increasingly viewed as an important target for the development of novel approaches to diagnose, prevent, or treat a wide range of disorders [1–4]. In this context, probiotics are among the most promising tools for manipulation of the gut microbiome, and have been defined as “live microorganisms which when administered in adequate amounts confer a health benefit on the host” [5]. The majority of probiotics are Gram-positive bacterial species, and considerable evidence is accumulating regarding the efficacy of these organisms in treating or preventing a variety of gastrointestinal (GI) diseases, and potentially also extra-intestinal disorders [1–4].

Among the probiotics currently available, *Escherichia coli* Nissle 1917 (EcN; serotype O6:K5:H1) is of particular interest. Not only is this one of the most extensively characterized probiotic organisms (in terms of phenotype, genotype, and clinical efficacy), but is currently the only Gram-negative species in use [6]. EcN was first isolated from the faeces of a World War I soldier who, in contrast to comrades in his trench, was not affected by an outbreak of dysentery [7]. This gastroprotective strain is the active component of Mutaflor (Ardeypharm GmbH, Herdecke, Germany), a microbial drug that is marketed and used in several countries. Clinical trials have shown EcN to be effective for maintaining remission of ulcerative colitis (UC) [8–11], stimulation of the immune system in premature infants [12], treatment of infectious diarrhoea [13], and protection of human intestinal epithelial cells (IECs) against pathogens [14, 15]. These benefits are largely attributed to the immuno-modulatory effects elicited by EcN, which encompass both innate and adaptive elements of the immune system. For example, colonisation with EcN has been indicated to alter the host cytokine profile, and also chemokine production in cultured IECs [16–19]; stimulate the production of mucosal peptide based defences [20]; influence the clonal expansion of T-Cell populations, and modulate antibody responses [12, 21, 22]. Notably, the modulation of T-cell functions mediated by EcN may also extend to γδ T-cells, potentially enabling EcN to coordinate modulation of both innate and adaptive responses [22]. EcN has also been indicated to alter COX-2 expression in intestinal epithelial cells [23], which is an important target in the treatment or prevention of several GI diseases including IBD and colorectal cancer [24–27].

Although most closely related to uropathogenic strains of *E*. *coli* (UPEC), EcN is considered non-pathogenic. Genomic characterisation has highlighted the absence of genes encoding the typical UPEC virulence factors, but the retention or accumulation of factors proposed to facilitate general adaptability, colonisation of the GI tract, and the probiotic effects of EcN [28, 29]. These include a range of surface associated structures that are likely to provide the primary interface between host and microbe in the GI tract, such as flagella, fimbriae, a special truncated lipopolysaccharide (LPS) variant, and a K5 type polysaccharide capsule [6, 29–31]. In particular, structures such as flagellin, peptidoglycan and LPS, are recognised by immune regulating Toll-like receptors (TLRS) expressed by IECs, which have been established as key routes of host-microbe communication in the gut, with TLR signalling integral to epithelial homoeostasis and defence [32–34]. Signaling by several TLRs is known to be modulated either directly or indirectly by EcN derived ligands [6, 17–20, 30, 35], which include surface associated structures absent in most or all other probiotic organisms. The K5 capsule produced by EcN in particular is notable in this context, and although not a ligand for known TLRs, the EcN capsule has been implicated in the interaction of this organism with IECs, and impact on chemokine expression and TLR signalling [18,19].

Nevertheless, as with other probiotics, the detailed mechanisms underlying the clinical effectiveness of EcN remain poorly understood overall, with a greater comprehension required to fully realise the potential of this important probiotic species. Here we describe the application of random transposon mutagenesis to identify genes and surface structures involved in the interaction of EcN with human intestinal epithelial cells, and provide new insight into the mechanisms through which EcN interacts with epithelial cells.

## Results

### Isolation and genetic characterisation of EcN mutants with disruptions in genes related to cell surface structures

Because cell surface structures are a primary point of contact between EcN and IECs, and processes such as biofilm formation and attachment to abiotic surfaces also depends on many of the same structures, we reasoned that selection of mutants with alterations in biofilm formation would enrich for those defective in cell surface associated features also likely to be involved in EcN-IEC interaction. Therefore, we initially subjected a total of 4,116 EcN mini-Tn*5* mutants to a preliminary high throughput screen for alterations in biofilm formation (both enhancements and reductions), in order to enrich for mutants attenuated in cell surface features. In this precursor biofilm screen 21 mutants were found to be significantly different in their ability to form biofilms as compared to the EcN wild-type (EcN WT), but unaltered in general growth rate. The majority of these (n = 15) exhibited a biofilm formation enhanced (BFE) phenotype, whereas six exhibited biofilm formation deficient (BFD) phenotype as compared to the WT (Table 1). Identities of genes disrupted in these mutants indicated that the majority were associated with synthesis of cell surface structures, or aspects of cell envelope biogenesis, previously linked to host-IEC interaction or intestinal colonisation (Table 1; [18, 35, 37–40]). A subset of 6 mutants disrupted in genes predicted to encode for cell surface structures, and encompassing both BFD and BFE phenotypes, were subsequently selected for further characterisation of their interaction with cultured IECs.

**Table 1:** Analysis of nucleotide sequence in EcN mutants with biofilm formation mutants.

Mutant ^a^	% of wild-type biofilm formation ^b^	Phenotype ^c^	Putative function/product of disrupted gene ^d^	
**JNBF1**	36 (0.6)	BFD	EcN CCQ07028.1: Arginine/ornithine antiporter ArcD	+
**JNBF2**	45 (0.5)	BFD	EcN CCQ07908.1: Type 1 fimbriae anchoring protein FimD	+
JNBF3	49 (0.3)	BFD	EcN CCQ05466.1: Flagellar hook-associated protein FliD	+
JNBF4	53 (0.9)	BFD	EcN CCQ05486.1: Flagellar motor switch protein FliM	+
**JNBF5**	55 (4.9)	BFD	EcN CCQ05465.1: Flagellar biosynthesis protein FliC	+
**JNBF6**	55 (0.8)	BFD	EcN CCQ07955.1: Periplasmic thiol:disulfide interchange protein DsbA	+
JNBF7	151 (3.6)	BFE	EcN CCQ06617.1: CidA-associated membrane protein CidB	+
JNBF8	157 (2.4)	BFE	EcN CCQ05558.1: Integrase	
JNBF9	157 (5.8)	BFE	EcN CCQ07966.1: Hypothetical protein.	
JNBF10	162 (0.8)	BFE	ECN CCQ05175.1: Hypothetical protein. *Similar to inner membrane transport protein ydiN from E. coli EPECa12 (1e-121, 200/225).	+
JNBF11	164 (7.5)	BFE	EcN CCQ05967.1: Hypothetical lipoprotein yghG precursor	
JNBF12	170 (5.9)	BFE	EcN CCQ05908.1: Uncharacterised protein YggN. *Similar to hypothetical protein ECG581_3391 from E. coli G58–1 (1e-67 105/105). Contains partial ansB/L-asparaginase II conserved domains (PRK11096, 2.44e-04)	
JNBF13	170 (3.2)	BFE	EcN CCQ05917.1: Putative inner membrane protein YqgA	+
JNBF14	171 (5.1)	BFE	EcN CCQ05004.1: Chaperone HdeA	
JNBF15	172 (2.5)	BFE	EcN CCQ06089.1: G:T/U mismatch-specific uracil/thymine DNA-glycosylase	
**JNBF16**	179 (6.6)	BFE	EcN CCQ05952.1: KfiB protein, from K5 biosynthesis gene cluster	+
**JNBF17**	182 (4.8)	BFE	EcN CCQ05954.1: Polysialic acid transport ATP-binding protein KpsT, from K5 biosynthesis gene cluster	+
JNBF18	188 (8.5)	BFE	EcN CCQ04927.1: Hypothetical protein. *Contains conserved domains from Type IV secretion system components (COG 3157, 1.24e-44) and HNH endonucleases (cd00085, 9.37e-03).	+
JNBF19	194 (5.0)	BFE	EcN: CCQ04506.1: Signal transduction histidine-protein kinase BarA	+
JNBF20	200 (2.7)	BFE	EcN CCQ07742.1: Probable transcriptional activator for leuABCD operon	
JNBF21	222 (1.2)	BFE	Intergenic region: Tn inserted between Peptidyl-prolyl cis-trans isomerase PpiA precursor (EcN CCQ04837.1) and TsgA protein homolog (EcN CCQ04838.1)—presumed to disrupt promotor regions.	+

^a^ Mutants in bold were selected for further characterisation (See Fig. 1 and associated text).

^b^ Biofilm formation was calculated as a % of the wild-type level quantified using CV staining assay. Results represent the mean of replicate experiments (n = 3) and figures in parentheses show the standard error of the mean. All mutants shown were significantly altered in biofilm formation compared to the wild-type (*P* < 0.05).

^c^ BFD—biofilm formation deficient; BFE—biofilm formation enhanced.

^d^ Putative functions of disrupted genes were primarily assigned based on the correlation of sequences flanking mini-Tn*5* inserts to the EcN draft genome sequence [28]. ~40 nt directly flanking the transposon were mapped to EcN contigs.

* In cases where EcN genome annotations did not provide a clear indication of function, BlastX searches against the full nr dataset, and/or the conserved domain database were used to infer function, with values in parentheses providing e-value and identity for these searches.

^+^ indicates genes predicted to be involved in synthesis of surface associated structures, broader aspects of cell envelop biogenesis, or associated with the cell envelop.

**Fig 1:**
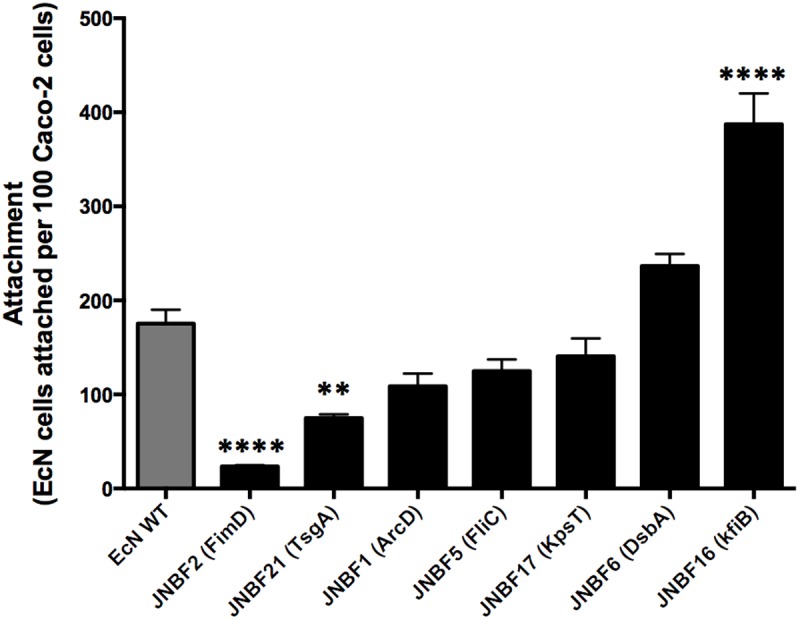
Adherence of EcN mini-Tn*5* mutants to Caco-2 cells. A subset of mutants recovered from biofilm screens with disruptions in genes predicted to be involved in generation of surface tstructures, were assessed for their ability to attach to Caco-2 cells in *in vitro* co-culture models. Caco-2 cell monolayers (~80% confluence) were exposed to bacterial suspensions from mid-log-phase cultures at an MOI of 1:1 for 4 h at 37°C, 5% CO_2_. Genes disrupted in mutants tested are noted in parentheses and details can be found in Table 1. Data are expressed as the mean of three replicates, and error bars show SE of the mean. Significant differences between attachment of EcN WT and mutants is indicated by ** (*P* ≤ 0.01) or **** (*P* <0.0001).

### Adherence of EcN cell surface structure mutants to Caco-2 cells

The ability of selected mutants to attach to intestinal epithelial cells was examined using an *in vitro* co-culture model, in which Caco-2 cells were exposed to EcN Tn mutants at a multiplicity of infection (MOI) of 1:1 (bacteria:Caco-2). Overall, the levels of adherence observed in these experiments showed no relationship with abilities to form biofilms on abiotic surfaces, but most mutants tested were observed to differ in levels of adherence compared to wild-type cells (Fig. 1). The lowest levels of adherence to Caco-2 cells were noted for mutant JNBF2, disrupted in the *fimD* gene of the type 1 fimbrial biosynthesis pathway (Table 1), in keeping with the established role of type 1 fimbriae in EcN host cell attachment [35]. In contrast, the greatest level of adherence to Caco-2 cells was observed for mutant JNBF16 (Fig. 1). This mutant was found to be disrupted in the *kfiB* gene, part of the K5 capsule biosynthesis gene cluster and vital for K5 production, indicating JNBF16 to be capsule deficient ([29, 41, 42], S1 Fig; Fig. 1). This *kfiB* mutant exhibited ~2.2-fold greater attachment to Caco-2 cells than the wild-type EcN. In contrast, it was notable that mutant JNBF17 was also disrupted in the *kpsT* gene associated with K5 capsule synthesis, but exhibited no significant alterations in adherence to Caco-2 cells (Table 1, S1 Fig). Since *kpsT* has previously been established to encode ABC transporter functions vital for K5 production [41, 43], this pointed to a role for *kfiB* specifically in the JNBF16 phenotype, rather than loss of the K5 capsule in general.

### Generation and characterisation of EcNΔ*kfiB* and EcNΔ*kfiC* mutants

**Fig 2:**
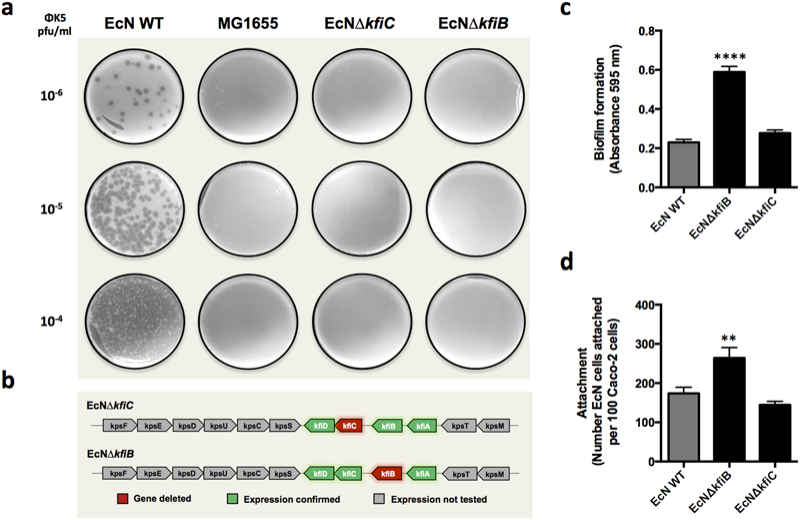
Initial characterisation of *kfiB* and *kfiC* deletion mutants. Isogenic mutants deleted for the K5 capsule biosynthesis genes *kfiB* (EcNΔ*kfiB*) or *kfiC* (EcNΔ*kfiC*) were assessed to confirm attenuation of capsule production, ensure gene deletions did not impact expression of downstream genes, and assess impact on biofilm formation and Caco-2 adherence. **A)** Loss of capsule production was confirmed using the ΦK5 bacteriophage sensitivity assay, in which cells lacking a K5 capsule are resistant. Images show results from soft ager overlay plates for strains exposed to 10^6^ to 10^4^ pfu/ml of bacteriophage, in which phage replication is manifest as plaques or clearing of the confluent bacterial growth. EcN WT—*E*. *col*i Nissle 1917 wild-type; MG1655—*E*. *coli* K12 control strain naturally lacking a K5 capsule; EcNΔ*kfiB*—EcN *kfiB* deletion mutant; EcNΔ*kfiC*—EcN *kfiC* deletion mutant. **B)** Expression of associated genes in the *kfi* gene cluster were assessed in deletion mutants using RT-PCR, and figures indicate gene found to be active in each mutant. **C)** The impact of *kfiB* or *kfiC* gene deletion on the ability of EcN to form biofilms on abiotic surfaces was assessed using the CV biofilm assay, as originally used to screen mini-Tn*5* mutants, and compared to the levels of WT biofilm formation. Data shows absorbance readings obtained following elution of CV stain from biofilms. **D)** The impact of *kfiB* or *kfiC* gene deletion on the ability of EcN to adhere to cultured Caco-2 cells was assessed using a co-culture system as for Fig. 1. EcN WT—*E*. *coli* Nissle 1917 wild-type; EcNΔ*kfiB*—EcN *kfiB* deletion mutant; EcNΔ*kfiC*—EcN *kfiC* deletion mutant. All figures show the mean of three replicate experiments, and error bars show SE of the mean. Significant differences between WT EcN and mutants is indicated by ** (*P* ≤ 0.01) or **** (*P* <0.0001).

The significant alterations in interaction of the JNBF16 mutant with Caco-2 cells was in contrast to previous reports of the interaction of capsule deficient EcN with Caco-2 cells, where no significant change in attachment was observed [18]. This prompted us to explore the role of *kfiB* in further detail, through the construction of two isogenic knockout mutants in which either *kfiB* (EcNΔ*kfiB*) or the downstream *kfiC* gene (EcNΔ*kfiC*) were deleted (S1 Fig). Attenuation of capsule biosynthesis in both mutants was assessed using the bacteriophage ΦK5 sensitivity assay, in which loss of K5 capsule results in resistance to ΦK5 infection [18, 44]. Both EcNΔ*kfiB* and EcNΔ*kfiC*, as well as the K12 control strain MG1655 were resistant to phage ΦK5, confirming capsule synthesis was compromised in both mutants (Fig. 2a). The expression of other genes in the region 2 *kfi* sub-cluster were also tested in both EcNΔ*kfiB* and EcNΔ*kfiC* using RT-PCR, and confirmed that gene deletions did not compromise expression of other *kfi* genes in either mutant (Fig. 2b). The original enhanced biofilm formation phenotype on abiotic surfaces was retained by EcNΔ*kfiB*, but despite the common attenuation in K5 capsule production, EcNΔ*kfiC* exhibited no alteration in biofilm formation on abiotic surfaces (Fig. 2c). EcNΔ*kfiB* also exhibited significantly greater levels of adhesion to Caco-2 cells compared with both the EcN wild-type and EcNΔ*kfiC* (Fig. 2d), in keeping with results from experiments with the original JNBF16 Tn mutant (Fig. 1). In contrast EcNΔ*kfiC* exhibited levels of adherence comparable to the wild-type (Fig. 2d).

### Impact of EcNΔ*kfiB* and EcNΔ*kfiC* on Caco-2 cell health

**Fig 3:**
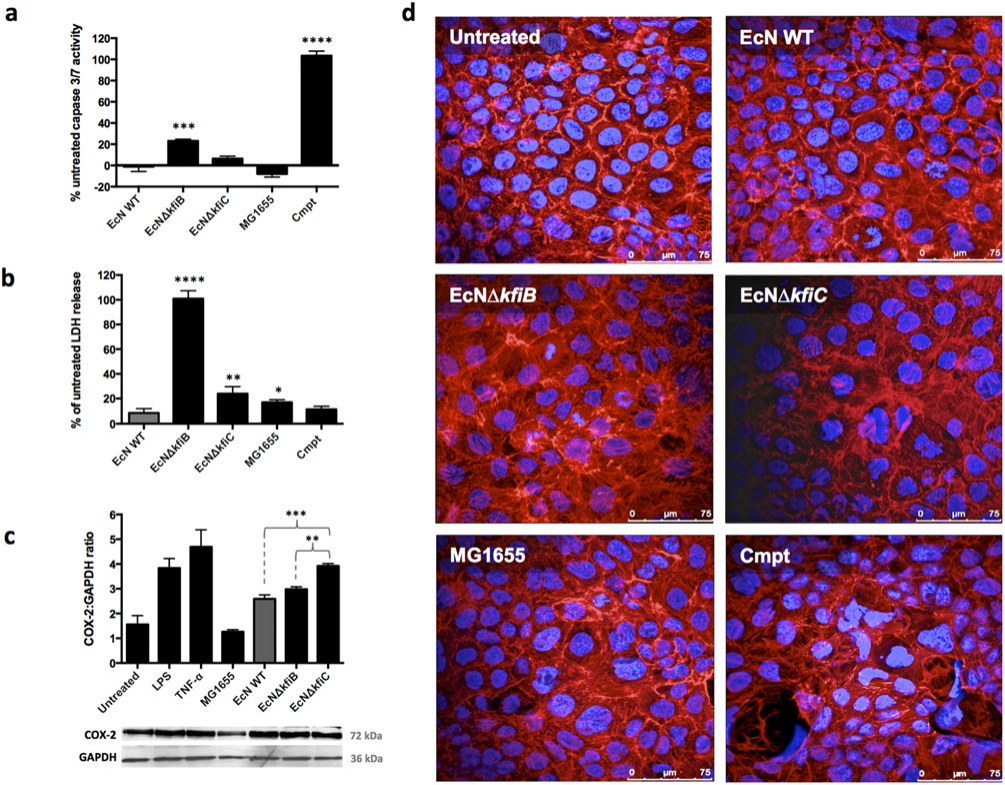
Impact of *E*. *coli* Nissle capsule deficient mutants on Caco-2 cell health. Owing to differences in adherence to Caco-2 cells, the impact of *kfiB* or *kfiC* gene deletion, and the K5 capsule on cell health was investigated using the co-culture system. **A)** The impact of capsule deficient mutants and wild-type strains on apoptosis was assessed by measuring caspase 3/7 activity after exposure to *E*. *coli* strains (MOI 10:1, bacteria:Caco-2) using the Caspase-Glo 3/7 luminescent assay. Cells treated with camptothecin (0.1 M) served as positive controls for apoptosis. Activity measured in relative light units (RLUs) and expressed as a % difference in activity observed in untreated Caco-2 controls. **B)** Cytotoxic effects of capsule deficient mutants were assessed by measuring the release of Lactate Dehydrogenase (LDH) from Caco-2 cells, after exposure to *E*. *coli* strains (MOI 10:1). LDH in media was quantified using the CytoTox 96 colorimetric assay (OD 490nm), and readings normalised to values obtained from complete lysates of untreated Caco-2 cells (100% LDH). Differences in LDH release between treatments was expressed as the % difference in normalised LDH release observed in media from untreated Caco-2 cells. **C)** The expression of COX-2 in lysates from treated or untreated Caco-2 cells was determined by Western blotting using anti-COX-2 antibody, followed by densitometry of developed films. Caco-2 cells were treated with *E*. *coli* cells from mid-log phase cultures at an MOI of 10:1, LPS, or human TNF-α; the latter treatments serving as positive controls for COX-2 expression. Quantities of COX-2 protein were normalised to densitometry readings from GAPDH. The chart provides normalised COX-2 densitometry readings, as the mean of three independent experiments, and error bars SE of the mean. Images show example blots for COX-2 and GAPDH. **D)** Changes in the actin cytoskeleton and nuclear morphology were assessed by double staining of treated and control cells with phalloidin red (F-actin) and DAPI (DNA). Stained cells were viewed using confocal laser microscopy, and images shown are representative of replicate experiments. Images were processed only to normalise brightness, contrast, and saturation. Data represent the means of 4 replicate experiments, and error bars show SE of the mean. EcN WT—*E*. *coli* Nissle 1917 wild-type; MG1655—*E*. *coli* K12 control strain naturally lacking a K5 capsule; EcNΔ*kfiB*—EcN *kfiB* deletion mutant; EcNΔ*kfiC*—EcN *kfiC* deletion mutant; Cmpt—Camptothecin (0.1 M). Significant differences to untreated controls are indicate by: * (*P* ≤ 0.05); ** (P ≤ 0.01); *** (*P* <0.001); **** (*P* <0.0001).

Distinct differences between EcNΔ*kfiB* and EcNΔ*kfiC* were also observed when effects on cell health were assessed. Compared to the EcN WT, EcNΔ*kfiB* significantly increased activation of caspase 3/7 (indicative of apoptosis), as well as release of lactate dehydrogenase (LDH) into the medium (indicative of general cell damage; Fig. 3a, b). In contrast, EcNΔ*kfiC* promoted no significant changes in levels of caspase 3/7 activation, and although exposure resulted in a significant increase in LDH release, this was considerably less pronounced than in cells exposed to EcNΔ*kfiB* (Fig. 3b). We also assessed the impact of these mutants on COX-2 protein expression, an important therapeutic target for probiotics in the GI tract. Compared to Caco-2 cells treated with EcN WT or EcNΔ*kfiB*, cells treated with EcNΔ*kfiC* expressed significantly greater levels of COX-2, which were comparable to those induced by the pro-inflammatory stimuli used as positive controls (*Salmonella* LPS and human TNF-α; Fig. 3c). When Caco-2 cells treated with either EcN WT, EcNΔ*kfiB* or EcNΔ*kfiC* were examined microscopically, following double staining of actin and nuclei (Phalloidin red and DAPI respectively), Caco-2 cells exposed to EcNΔ*kfiB* were observed to exhibit greater incidence of condensed chromatin and nucleus defragmentation, as well as rearrangements in the actin cyctoskeleton. However, these features were not evident in cells exposed to EcN WT or MG1655, and considerably less pronounced in Caco-2 cells treated with EcNΔ*kfiC* (Fig. 3d). Co-culture with supernatants from EcNΔ*kfiB* did not induce effects observed when cell suspensions were used in co-culture models, suggesting that bacterial contact was essential for the observed effects.

### Assessment of EcN morphology and aggregation

**Fig 4:**
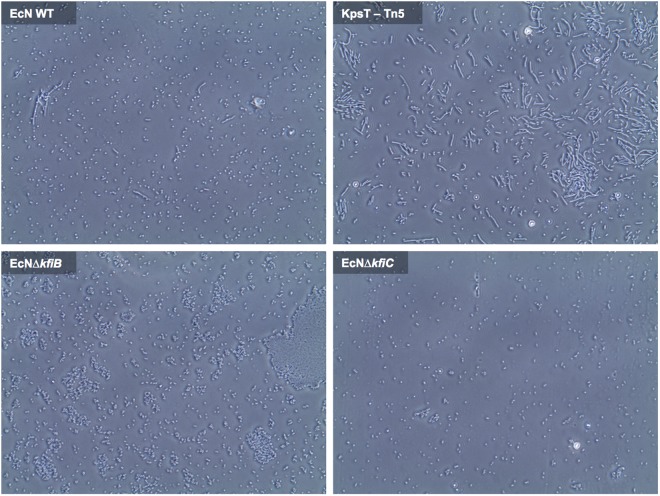
Cell morphology and formation of aggregates in EcN wild-type and K5 capsule mutants. The potential for alterations in cell-cell interaction, and the general cell morphology of EcN WT and capsule mutants was examined by phase contrast microscopy. 16h cultures were examined directly by phase contrast microscopy, to assess cell morphology and the potential for cellular aggregation in EcN WT and capsule mutants. 9 fields of view were selected at random for each slide examined, and subsequently reviewed as a collection to identify the pervading features. Images provide representative examples from assessment of each strain. EcN WT—*E*. *coli* Nissle 1917 wild-type; KpsT-Tn*5*—EcN JNBF17 mini-Tn5 mutant disrupted in the K5 *kpsT* gene (Table 1); EcNΔ*kfiB*—EcN *kfiB* deletion mutant; EcNΔ*kfiC*—EcN *kfiC* deletion mutant. Images are at 40× magnification and represent entire fields of view.

Mutants lacking an outer polysaccharide capsule may also be altered in interactions between bacterial cells, which could contribute to the observed phenotypes of capsule mutants and their effects on Caco-2 cells. Therefore, using phase contrast microscopy, we examined cell suspensions from cultures of EcNΔ*kfiB* and EcNΔ*kfiC*, and compared these with the EcN WT and the *kpsT*::mini-Tn*5* mutant JNBF17. This latter mutant was isolated in original biofilm screens (Table 1), and was included here since it is also blocked in K5 capsule production (based on resistance to ΦK5—S1 Table), but at a distinct stage in K5 assembly compared to EcNΔ*kfiB* and EcNΔ*kfiC* (mature polysaccharide transport to the cell surface in *kpsT*::mini-Tn*5*; S1 Fig). In all mutant strains distinct differences compared to the wild-type were noted in terms of cell morphology and the presence of cellular aggregates. However, each mutant also displayed a distinct phenotype when compared with all others (Fig. 4). Wild-type cultures were found to be composed predominantly of short vegetative cells, with only few small aggregates observed, but a consistent low level of highly elongated cell forms were present (Fig. 4). In the JNBF17 *kpsT* mutant, the incidence of elongated cells was considerably greater, and these were frequently observed to form large, seemingly loosely associated aggregates (Fig. 4). In contrast, both EcNΔ*kfiB* and EcNΔ*kfiC* cultures were characterised by a complete absence of elongated cell forms and were manifest as very short vegetative cells. However, in contrast to EcNΔ*kfiC*, cells from EcNΔ*kfiB* were found to frequently form large aggregates, apparently with tight association between participating cells in many instances (Fig. 4).

## Discussion

In this study we applied random transposon mutagenesis to identify genes relevant to EcN-IEC interaction. This strategy was selected as it is not influenced by existing information, and makes no prior assumptions regarding the involvement of particular genes in a given trait or process. This approach identified the *kfiB* gene, and by inference K5 capsule biosynthesis (S1 Fig), to be important for EcN interaction with Caco-2 cells. Although the role of the K5 capsule in EcN-IEC interaction has been previously investigated, these studies utilised mutants decapsulated *via* deletion of the *kfiC* gene, and the increased adherence displayed by the JNBF16 *kfiB* mutant is in stark contrast to findings from these previous studies, which showed no alteration in adherence to Caco-2 cells in *kfiC* mutants [18, 19]. Characterisation of a markerless mutant deleted in *kifB* (EcNΔ*kfiB*) supported results obtained with the initial JNBF16 mini-Tn*5 kfiB* mutant, eliminating the possibility of factors relating to Tn mutagenesis to have led to a spurious phenotype in this mutant, and confirmed a role for *kfiB* in EcN adherence to Caco-2 cells.

However, since the *kfiB* product presently has no clearly defined role in K5 capsule biosynthesis, and loss of K5 capsule had not previously been associated with the JNBF16 phenotype in EcN, we also constructed an isogenic mutant deleted in the downstream *kfiC* gene (EcNΔ*kfiC*) as a comparator. In contrast to *kfiB*, the function of *kfiC* in capsule formation has been clearly established, and loss of this glycolsyltransferase blocks synthesis of capsule precursors in the cell cytoplasm, and ultimately assembly of the K5 capsule [18, 36]. Although the abolition of capsule biosynthesis through deletion of closely related genes in the K5 pathway would be expected to yield mutants with comparable phenotypes, and both EcNΔ*kfiB* and EcNΔ*kfiC* were indicated to be capsule deficient in the ΦK5 bacteriophage assay, the phenotypes of these mutants were distinct in almost all other assays (Summarised in S1 Table). Collectively, our data suggest that loss of distinct K5 *kfi* genes may result in mutants with differing impacts on Caco-2 cells in co-culture experiments. More specifically, we found that loss of *kfiC* leads to increased COX-2 expression in epithelial cells treated with this EcN mutant, while the loss of *kfiB* leads to increased adherence, cytotoxicity, and induction of apoptosis (S1 Table).

The reasons for the differing impact of EcNΔ*kfiC* and EcNΔ*kfiB* on Caco-2 cells is currently unclear, but may relate to wider effects on the complex protein-protein interactions underlying K5 polysaccharide formation, as well as the loss of specific functions encoded by disrupted genes. The K5 polysaccharide precursors are synthesised by the glycosyl transferases encoded by *kfiA* and *kfiC*, which work in a coordinated fashion to link repeating units of GlcNAc (KfiA) and GlcA (KfiC) from UDP sugar precursors; with KfiD providing substrates for KfiA through isomerisation of UDP-Glc to UDP-GlcA, [S1 Fig; Reviewed in Whitfield [41], Corbett and Roberts [45], Whitfield and Roberts [46]]. This process also requires the formation of KfiABC membrane associated complexes, for the coordinated enzyme activity necessary for K5 precursor production and export (S1 Fig; [36, 44, 47]). Of particular relevance to our observations are findings that loss of KfiB prevents membrane localisation of KfiC, but not of KfiA [44]. In contrast, loss of KfiC abolishes the localisation of KfiA as well as KfiB [44]. This hierarchical interaction of Kfi proteins, and the associated model for K5 biosynthesis, also predicts EcNΔ*kfiB* mutants to retain KfiA activity but exhibit significant reduction or loss of KfiC activity, due to abolished membrane localisation of this protein. In contrast, EcNΔ*kfiC* would be predicted to exhibit complete disruption of the K5 polysaccharide biosynthetic complex, since the deletion of *kfiC* also leads to abolished membrane targeting in KfiA and KfiB [44].

Thus, it may be hypothesised that EcNΔ*kfiC* could be subjected to a more complete disruption of the K5 biosynthesis machinery, while EcNΔ*kfiB* may retain the capacity for reduced or aberrant K5 precursor synthesis. This in turn raises the possibility that the divergent phenotypes observed in these mutants arise from distinct differences in the assembly of surface structures related to capsule biosynthesis. Notably, the current model of K5 biosynthesis described above predicts EcNΔ*kfiB* to still be theoretically capable of polymer synthesis, albeit at greatly reduced efficiency, since the key glycosyl transferases (KfiA, KfiC) as well as UDP-glucose dehydrogenase KfiD are still generated. Available evidence also suggests the K antigen export machinery (KpsMTDE; S1 Fig) does not exhibit specificity for the polysaccharide chain, and is suggested to instead be specific for the kdo lipid modification [41], an aspect predicted to be unaffected in EcNΔ*kfiB* or EcNΔ*kfiC*, and permissive of the potential for altered capsule components to be exported.

Although the synthesis of partial or aberrant K5 structures through loss of specific genes has not previously been reported in such mutants, it is notable that many studies seeking to identify and characterise genes essential for K5 biosynthesis have used resistance to the ΦK5 bacteriophage as a sole indicator of capsule loss [18, 19, 36, 44]; as has also been deployed in the present study. However, while resistance to ΦK5 is generally taken to be synonymous with a complete absence of any K5 capsule structure, this does not preclude the assembly of a reduced capsule structure, or the decoration of the cell surface with aberrant or partial K5 components. Studies of capsule biosynthesis in the related K1 system (also group 2 capsular polysaccharides), and the interaction of associated phage have demonstrated that K1 capsules with altered structure may be produced but are resistant to phage K1 infection [48]. Furthermore, alterations in the overall level of capsule production has also been found to influence phage infection in K1 strains, with mutants that exhibit structurally normal but reduced capsule production resistant to phage infection [49].

Therefore, while the ΦK5 assay can provide a gross indication of perturbation in capsule biosynthesis or assembly, it is unlikely to provide insight into more subtle alterations in surface features that may be manifest in the K5 mutants studied here, such as reduced levels of K5 synthesis, or the presentation of structurally abnormal capsule components on the cell surface. Such differences could lead to the distinct phenotypes displayed by these mutants with respect to biofilm formation and interaction with Caco-2 cells, yet would be in keeping with a common phenotype in ΦK5 assays. When the data from K5 deficient mutants generated in this study are considered collectively, and with respect to the potential limits of the ΦK5 assay in resolving the finer detail of capsule attenuation, the most parsimonious explanation for the divergent phenotypes would seem to be the continued production of at least some K5 associated factors in one or both of these mutants (S1 Table).

The potential for differences in the specific level or nature of K5 decapsulation in EcNΔ*kfiB* or EcNΔ*kfiC*, also raises the possibility for involvement of other cell surface moieties in the impact of these mutants on cultured epithelial cells. In terms of the EcNΔ*kfiB* phenotype, factors that promote auto-aggregation and biofilm formation in *E*. *coli*, would be prime candidates. These include the Ag43 and AidA auto-transporter adhesins found in numerous strains of *E*. *coli*, and for which homologues have been identified in the EcN genome [29, 50, 51]. This theory is further supported by studies demonstrating the expression of a colanic acid capsule, or K5 capsule, to produce a masking effect for auto-transporter adhesins like Ag43 and AidA in some strains, effectively shielding these surface factors, and blocking their contribution to biofilm formation as well as adherence to cultured human cells [50, 52].

In this scenario, the increased adherence of EcNΔ*kfiB* to Caco-2 cells may be the result of enhanced EcN auto-aggregation and self-recognition, with attached EcNΔ*kfiB* cells acting as foci for further attachment and accumulation of planktonic EcN. The elevated biofilm formation ability, increased formation of cellular aggregates, and enhanced attachment to host cells evident in the EcNΔ*kfiB*, is congruent with this hypothesis, as well as the lack of activity observed from cell free supernatants. In contrast, the phenotype of the *kpsT*::mini-Tn5 mutant (JNBF17; Table 1, S1 Fig) would seem to only partially support this unmasking and auto-aggregation hypothesis. KpsT encodes for a component of the K5 polysaccharide transport machinery, and mutations in this gene have been shown to result in intra-cellular accumulation of polysaccharide capsule precursors and lack of detectable capsule expression [43]. Therefore, if unshielded surface proteins were a factor in the EcNΔ*kfiB* phenotype, the JNBF17 *kpsT*::mini-Tn5 mutant would be expected to exhibit a comparable behaviour in the same assays. However, while JNBF17 showed an increase in biofilm formation, cellular aggregation, and was also resistant to the K5 bacteriophage, this did not translate to significant increases in adherence to Caco-2 cells.

These results do not preclude EcNΔ*kfiB* decapsulation from promoting auto-aggregation, which is still supported by the JNBF17 phenotype and other data, but indicates the requirement for additional factors in Caco-2 cell adherence specifically. The intracellular location of KpsT precludes a direct role for this protein in adherence (S1 Fig), again indicating the adherent phenotype of EcNΔ*kfiB* is mediated by factors, presumably related to K5 synthesis, that are still exported in this mutant. This is also consistent with the theoretical impact of *kfiC* or *kfiB* deletion on assembly of the biosynthetic machinery, and the prediction of a more substantive impact from loss of *kfiC*. Alternatively, the potential for the alterations in JNBF17 cell morphology to play a role in the lack of increased adherence to Caco-2 cells cannot be fully excluded, but since JNBF17 still retained levels of adherence comparable to the WT it seems most likely not to play a significant role.

Overall the evidence presented here, supports a number of theories regarding the divergent phenotypes of EcNΔ*kfiC* and EcNΔ*kfiB*, but it is clear that further work will be required to firmly establish the mechanisms through which loss of *kfiB* or *kfiC* lead to such different interactions with IECs. In particular, the potential for wider aspects of cell physiology to be affected by perturbation of K5 synthesis is suggested by the mutants characterised in this study. For example, formation of elongated cells in JNBF17 point to an impact on regulation of broad aspects of cell replication and cell division. Therefore, the perturbation of K5 capsule biosynthesis seems likely to have considerable impact on fundamental cellular processes, and opens the potential for the observed effects on Caco-2 cell health to relate to disruption of wider cellular functions, rather than aspects of K5 capsule assembly alone.

Nevertheless, our results reinforce the importance of the K5 capsule in facilitating the interaction between EcN and IECs, and indicate the potential for distinct differences in host-microbe interaction to be mediated by modulation of K5 capsule production. This highlights the possibility that alterations in capsule structure may govern a range of possible outcomes arising from host-EcN interaction. Further studies, utilising a more definitive array of mutants in K5 biosynthesis genes, stand to provide much fundamental insight into the mechanisms underpinning the beneficial effects of EcN and its interaction with the host epithelium. Furthermore, as a well-characterised probiotic with proven clinical effect, for which a broad array of robust genetic tools are available, EcN is a prime candidate for the development of rational approaches to probiotic design and tailored disease interventions. The results of this and subsequent studies will contribute to the basic understanding required to underpin the rational design and development of such tailored probiotic interventions, which hold much promise for the prophylaxis of a variety of disorders, and the enhancement of human health.

## Materials and Methods

### Bacterial strains and cultures

*E*. *coli* Nissle 1917 wild-type (EcN WT) (Ardeypharm GmbH, Herdecke, Germany) and *E*. *coli* K12 MG1655 wild-type (CGSC7740) were used in this study. *E*. *coli* BW29427, diamonopimelic acid auxotroph and:: *pir* (Datsenko KA and Wanner BL, Purdue University) served as the mini-Tn*5* donor strain. All strains were grown in Luria-Bertani (LB) (Fisher Scientific, UK) at 37°C with shaking. Media were supplemented with 50 μg ml^–1^ kanamycin (Km) for culture of mini-Tn*5* transposon donor strain, 30 μg ml^–1^ kanamycin and 100 nM diamonopimelic acid (DAP) (Sigma-Aldrich, UK) for BW29427. Where appropriate the growth media was supplemented with 20 μg ml^–1^ chloramphenicol (Cm), 12 μg ml^–1^ tetracycline (Tc), or 200 μg ml^–1^ gentamicin (Gen) (Sigma, UK).

### Random transposon mutagenesis

Mutants were generated from EcN WT with the pRL27::mini-Tn*5* delivery system encoding a hyperactive Tn*5* transposase and carrying a mini-Tn*5* element, essentially as described by Larsen *et al*. [53]. Vector pRL27 was transferred to EcN WT from donor *E*. *coli* BW29427 by conjugal transfer. Mating experiments were performed with a donor/recipient ratio of 1/10, and the mixed cultures incubated for 8 h at 37°C on LB agar plates supplemented with 100 nM DAP and 10 mM MgSO_4_. Trans-conjugant EcN colonies were selected and subsequently cultured on LB agar supplemented with 50 μg ml^–1^ Km only. Mutants were stored in 96-well plates at -80°C until required.

Loss of the pRL27 delivery vector and the presence of random, single mini-Tn*5* chromosomal inserts were verified by analysis of restriction digest of plasmid extracts obtained using the QIAprep Miniprep kit (Qiagen, UK), and Southern hybridisation with a mini-Tn*5* specific (DIG)-labelled probe. Probes were generated by PCR amplification of the mini-Tn*5 nptII* gene with DIG-labelled dNTPs, components of the DIG luminescence detection kit for nucleic acids (Roche, Burgess Hill, UK), using primers NPT2_F1/R1 (S2 Table).

### Isolation of biofilm formation mutants

Mutants with altered biofilm forming abilities were isolated using the crystal violet (CV) staining-based assay based on that described by O’Toole and Kolter [54]. For the first screening step, mutants were inoculated in 100 μl LB broth per well (in 96 well plates), sealed with plastic films, and incubated statically at 37°C for 24 h. After incubation, cell cultures were decanted and wells were washed twice with sterile deionised water (SDW) to remove non-adherent cells, allowed to dry at room temperature (RT) for 5 min, then biofilms stained with 120 μl of a 1% CV solution per well (Pro-Lab Diagnostics, Neston, UK) for 15 min at RT. Following removal of excess CV, wells were washed three times with 150 μl sterile distilled water (SDW) to remove excess stain, before elution of cell-bound CV by addition of 150 μl DMSO. Optical density was then measured by spectrophotometry at 595 nm (OD_595_). The recorded readings were used to calculate the average OD_595_ of all mutants per plate. To detect mutants with potential alterations in biofilm formation, the pre-calculated average OD_595_ per plate was compared to individual readings, and those showing +/- 0.1 of the plate OD_595_ average (Biofilm enhanced or Biofilm deficient) were selected for the second-round screening.

The second screening step was performed to confirm the observed biofilm formation phenotypes of mutants isolated in first-round screens. The mutants were compared with the wild-type EcN for biofilm formation in 96-well plate, again using the CV staining assay as described above. For this screen, each plate contained wells inoculated with mutants (n = 4), the wild-type (n = 4), and uninoculated LB broth (n = 4), and was performed in triplicate. Prior to the biofilm staining, mutants were first assessed for their ability to grow as compared to the wild-type. Growth was measured by spectrophotometry at 600 nm (OD_600_). Mutants showing statistically significant differences in biofilm formation (at a *P* value ≤ 0.05), but without significant differences in bacterial growth (*P* value > 0.05), were confirmed biofilm altered mutants and defined as biofilm enhanced (BFE) or biofilm deficient (BFD) mutants. Mutants biofilm formation index was calculated as the percentage of CV (OD_595_) measured in the EcN WT.

### Genetic characterisation of biofilm-altered mutants

Genes disrupted in mutants of interest were identified using a “cloning free” arbitrary PCR-based approach to amplify DNA segments flanking the transposon insertion, as described by Manoil [55] using primers listed in S2 Table. The resulting amplicons were sequenced by GATC Biotech Ltd. (London, UK) using transposon end primer pLR27Primer 3. The putative function of disrupted genes was assigned by mapping sequence data flanking the mini-Tn*5* insert site to the *E*. *coli* Nissle Draft genomes sequence [28], and the previously published genomic islands [29]. Sequence reads from mutants were trimmed to remove the 5’ low quality regions (typically ~30–50 nt), and the immediate ~40 nt flanking sections correlated with the EcN genome. Where EcN genome annotations did not provide any clear indication of putative function wider searches of the nr dataset using BlastX and/or the conserved domain database were employed.

### Construction of *kfiB* and *kfiC* deletion mutants

Deletion mutants EcNΔ*kfiB* and EcNΔ*kfiC* were constructed by homologous recombination using the Xer-cise^TM^ chromosomal modification system (Cobra Biologics, Keele, UK) according to manufacturer’s instructions and protocols described by Bloor and Cranenburgh [56]. The system comprises plasmids pTOPO-DifCAT and pLGBE, for construction of target gene specific integration cassette and provision of the Red λ recombination functions, respectively. Briefly, *kfiB* or *kfiC* integration cassettes consisting of the *dif*_*E*. *coli*_-*cat*-*dif*_*E*. *coli*_ region from pTOPO-DifCAT plasmid flanked by 50 nt regions homologous to the 3’ and 5' ends of the target gene, were generated by PCR using 70-nt primers, *kfiB*.int_F/R or *kfiC*.int_F/R (listed in S2 Table). EcN WT was first transformed with the Tc-selectable plasmid pLGBE and transformants EcN-pLGBE were used to generate electrocompetent cells, which were subsequently transformed with the PCR product of the *dif*_*E*. *coli*_-*cat*-*dif*_*E*. *coli*_ integration cassette constructs. Integrants were selected on LB agar supplemented with 20 μg ml^–1^ Chloramphenicol. Loss of pLGBE and generation of chloramphenicol-sensitive clones, indicating resolution of *dif*_*E*. *coli*_-*cat*-*dif*_*E*. *coli*_ marker genes by native recombinases and generation of markerless deletion mutants (mutants EcNΔ*kfiB* and EcNΔ*kfiC*) was achieved by sub-culturing the integrants in LB broth in the absence of antibiotics. Loss of pLGBE was verified by plasmid extraction, and by PCR for marker cassettes *kfiB* or *kfiC* specific primers EcN*kfiB* _F/R or *E*cN*kfiC* _F/R, respectively, and confirmed by PCR.

### Examination of polar effects in EcNΔ*kfiB* and EcNΔ*kfiC* mutants

The effect of gene deletion or disruptions in *kfiB* and *kfiC* mutants, on the expression of downstream genes (polar effects) was assessed using RT-PCR. Total RNA was extracted from mid-log-phase bacterial cells using the RNeasy Protect Cell Mini Kit (Qiagen) according to manufacturer’s instructions, and treated using the Ambion TURBO DNA-*free* system (Ambion-Life technologies, Paisley, UK) to remove any potential DNA contamination. The treated RNA was used to generate cDNA using the One Step RT-PCR kit (Qiagen) according to the manufacturer’s instructions, utilising 15 ng RNA per reaction as template. Resulting cDNA was used as template in standard PCRs for detection of gene transcripts with specific primers detailed in S2 Table.

### Confirmation of K5 capsule absence in EcNΔ*kfiB* and EcNΔ*kfiC* mutants

The K5 capsule-specific bacteriophage (ΦK5) [57] was used in this study to determine if the K5 capsule was expressed by EcN WT and deletion mutants. The bacteriophage was diluted and maintained in phage dilution buffer (PDB) (100 mM NaCl, 8 mM MgSO4, 0.01% gelatine, 50 mM Tris pH 7.5). Cultures of mutants EcNΔ*kfiB* and EcNΔ*kfiC*, controls EcN WT and *E*.*coli* MG1655 were grown in LB with shaking at 37°C to an OD_600_ of 0.3 then pelleted by centrifugation (10,000 × *g* for 10 min) and resuspended in ice-cold 10 mM MgSO4. Aliquots of cell suspension (100 μl) were mixed with 100 μl of the appropriate bacteriophage dilution (ranging from 10^1^ to 10^9^ PFU ml^–1^ from stock suspension of 2.1 × 10^9^ PFU ml^–1^) in sterile 1.5 mL Eppendorf tube then incubated at RT for 30 min, statically. The phage-bacteria mixture was added to a volume of 3 ml of soft agar (1% NaCl, 0.5% yeast extract, 1% tryptone, 0.75% agar) held at 42°C in 15 ml sterile glass tube, and the content of the tubes were mixed gently by swirling. The inoculated soft agar was poured on top of LB agar and incubated for 16 h at 37°C to allow formation of plaques.

### Intestinal epithelial cell culture and co-culture conditions

Caco-2 cells (passage 51–79) were grown at 37°C with 5% CO_2_ in Dulbecco's modified Eagle's medium (DMEM, 4.5 g glucose l^–1^) supplemented with 10% fetal bovine serum and 1× non-essential amino acids (PAA Laboratories, Somerset, UK). Cells were seeded into 6-well or 96-well plates, grown up to ~ 60–80% confluence, and used in co-culture experiments with bacteria. Mid-log-phase bacteria (OD_600_ of 0.5) were washed with PBS and suspended in DMEM to the required final count, corresponding to the appropriate multiplicity of infection (MOI) and added to Caco-2 monolayers before plates were incubated at 37°C and 5% CO_2_.

### Bacterial adherence to Caco-2 cells

Adherence was calculated according to the strategy employed by Hafez *et al*. [18]. Mid-log phase bacteria cultures were suspended in DMEM then added to monolayers of Caco-2 grown in 6-well plates (80% confluence) at an MOI of 1:1 and incubated at 37°C and 5% CO_2_ for 4 h. The monolayers were washed 3 times with PBS to remove non-adherent cells then treated with lysis solution, 1% wt / vol saponin (Sigma Aldrich) in trypsin-EDTA (PAA Laboratories, Somerset, UK) for 10 min to allow permeabilisation of Caco-2 cells and recovery of total cell-associated bacteria. Cells were mixed gently by pipetting, serially diluted in sterile PBS, plated onto LB agar, and incubated at 37°C overnight. The obtained viable count represented the total number of cell associated bacteria (adherent and internalised). Internalised bacteria were calculated using the same protocol but Caco-2 cells were treated with gentamicin for 2h (200 μg ml^-1^) to kill external bacteria prior to lysis and enumeration. The number of adherent bacteria was taken as the difference between total cell associated bacteria and internalised bacteria.

### The effect of EcN mutants on induction of apoptosis in Caco-2 cells

The effect of EcN mutants on induction of apoptosis Caco-2 cells was assessed by measuring the activity of caspase 3/7 using the Caspase-Glo 3/7 kit (Promega, Southampton, UK), according to manufacturer’s instructions. Cells were seeded in 96-well plates with 5,000 cells/well and cultured to achieve ~ 60% confluence then treated with bacteria or bacterial supernatants in co-culture. Media was replaced with serum-free DMEM for 12 h prior to the treatment. Bacterial suspensions were prepared in serum-free DMEM from mid-log-phase cultures then added to Caco-2 cells at an MOI of 10:1 (bacteria:Caco-2) in a final volume of 100 μl/ well. The plates were incubated for 2 h at 37°C and 5% CO_2_ then media was replaced with fresh serum-free DMEM supplemented with gentamicin at 200 *μ*g ml^–1^ to stop bacterial growth, and plates were incubated for another 10 h. Bacterial supernatants were obtained from cells grown in 5 mL serum-free DMEM at 37°C overnight, with shaking, and recovered by centrifugation (1,500 × *g* for 10 min), pH adjusted to 7.2, and filter-sterilised (0.2μm). The supernatants were diluted in fresh serum-free DMEM at a ratio of 1:1, and used in place of cell suspensions as described above. Caspase 3/7 activity was measured as relative light units (RLUs) using a Synergy Multi-Mode Plate Reader (BioTek, Potton, UK) operated with BioTek Gen5.20 software.

### Analysis of cytotoxicity

The effect of EcN strains on induction of cytotoxicity in Caco-2 cells was assessed by measuring the amount of lactate dehydrogenase (LDH) released into the co-culture media, using the CytoTox 96 Non-Radioactive Cytotoxicity Assay kit (Promega). Caco-2 cells were treated with bacteria and controls as described for the analysis of apoptosis (above) and both assays were performed in parallel. After treatment of Caco-2 cells, supernatants were collected from plate wells using a multichannel pipette then transferred to fresh 96-well at 50 μl/well. The supernatant was diluted further in serum-free culture media then mixed with the CytoTox 96 substrate at a ration of 1:1. Plates were incubated in the dark at room temperature for 30 min and absorbance at 490 nm (OD_490_) was recorded. The percentage of cytotoxicity was calculated as LDH released in treated cells (OD_490_)/maximum LDH release (OD_490_) × 100. Maximum release was determined as the amount released by total lysis of untreated Caco-2 cells with the CytoTox 96 lysis Solution (10X).

### Analysis of cellular and nuclear morphology

Membrane integrity and nuclear morphology of Caco-2 cells were analysed by fluorescent *phalloidin* (*F*-*actin*) and Dapi (DNA) stainings. Cells were grown on sterile glass cover slips in 6-well plates then treated with EcN strains and controls (MG1655 and 0.1 mM camptothecin; Sigma) as described above (analysis of apoptosis). After the treatments, the cells on coverslips were washed with PBS then fixed with 4% formaldehyde (Sigma) in PBS for 20 min at RT. The fixed cells were washed three times with PBS and permeabilised with 0.1% Triton X-100 (Sigma) in PBS for 5 min at RT. The cells were washed three times with PBS, 5 min per wash with gentle rocking, then treated with a 0.1 μg ml^–1^ solution of fluorescein isothiocyanate-phalloidin (Sigma- Aldrich) in PBS for 1 h at RT in the dark. The cells were washed twice with PBS and were mounted with the Fluoroshield DAPI medium (Sigma) and examined under a Leica TCS SP5 Confocal Laser Scanning microscope (Leica Microsystems, Wetzlar, Germany).

### Analysis of COX-2 expression

The expression of COX-2 protein in Caco-2 co-cultures was analysed by western blotting using standard methods. Briefly, Caco-2 cells were seeded in 6 wells plates, and at ~ 60% confluence, were treated with EcN K5 mutants and controls as described above (analysis of apoptosis). Lipopolysaccharide (LPS, final concentration, 5 μg ml^–1^) from *Salmonella enterica* (Sigma, UK) and human tumour necrosis factor alpha (TNF-α, 10 ng ml^–1^) (Sigma, UK) were used as pro-inflammatory stimulator positive controls. Treated Caco-2 cell monolayers were washed 3 times with PBS, trypsinised then resuspended in 100 μl of hypotonic buffer (10 mM HEPES, 10 mM KCl, 0.1 mM EDTA, 0.1 mM EGTA, 1 mM DTT in SDW, pH 8.0), containing Sigma protease inhibitor cocktail (1:20), for 15 min at 4°C. Cells were lysed in 25 μl 10% Triton X-100 for 30 min and total protein obtained by centrifugation (10,000 g for 1 min at 4°C). Protein concentration was determined by the Bradford method (Bio-Rad) and equivalent amounts of protein lysates (10 μg) separated by electrophoresis on SDS—PAGE (10%), and then transferred onto a nitrocellulose membrane (GE Healthcare, Giles, UK). The blots were blocked at RT with 10% skimmed milk powder in TBST buffer (10 mM Tris, pH 7.6, 0.5 M NaCl, 0.05% Tween 20), and incubated with primary antibody, anti-COX-2 rabbit polyclonal (Abcam, Cambridge, UK) 1:1,000 in TBST, overnight at 4°C. Blots were washed with TBST then incubated with anti-rabbit HRP-conjugated secondary antibody (Sigma, UK) 1:5,000 in TBST, for 1h at RT. Membranes were washed further then visualised by incubation with the ECL chemiluminescent reagent (Amersham, Little Chalfont, UK) and exposed to Kodak Image Station 440 for signal detection. Blots were then stripped and reprobed with loading control anti-GAPDH mouse monoclonal (Ambion, Cambridge, UK); anti-mouse IgG HRP-conjugated (Sigma, UK) as secondary antibody. The bands of COX-2 densitometry readings were normalized to the GAPDH control.

### Analysis of cell morphology and aggregation

Bacteria were grown statically in 5 mL LB in 50 mL sterile polystyrene tube at 37°C for 16 h. The cultures were mix gently by swirling and 3 μL of each was directly transferred onto glass slide, allowed to rest for 1 min then covered with a cover slip and visualised using ×40 magnification phase contrast microscopy. For each culture 10 randomly selected fields of view across each slide were captured using the Olympus Cell Sense software, and subsequently reviewed. Representative images were selected and adjusted only for brightness and contrast.

### Statistical analysis

All statistical analysis was performed using Prism 6.0c For Mac OS X (Graphpad Software inc. USA; www.graphpad.com). Data was analysed using either Student’s *t-test*, or ANOVA with the Bonferroni correction for multiple comparisons.

## Supporting Information

S1 Fig Overview of K5 capsule biosynthesis in *E*. *coli*, and associated genes disrupted in this study. Diagrams show the genetic organisation of the K5 gene cluster in *E*. *coli* Nissle 1917 based on data from Cress *et al*. [28]; Grozdanov *et al*. [29], and an overview of the current model for K5 capsule biosynthesis and assembly adapted from Griffiths *et al*. [36]; Whitfield [41]; Petit *et al*. [42]; Bliss *et al*. [43]; Hodson *et al*. [44]; Corbett and Roberts [45]; Whitfield and Roberts [46]; Rigg *et al*. [47]; Whitfield and Willis [58]. **A)** Physical map of the EcN K5 capsular polysaccharide gene cluster. Region I (*kpsF*,*E*,*D*,*U*,*C*,*S*) and Region III (*kpsM*,*T*) encode elements of synthesis and export machinery, and are conserved among *E*. *coli* strains generating Group 2 polysaccharide capsules. Region II encodes K5 specific polysaccharide synthesis machinery (*kfiA*,*B*,*C*,*D*). Genes disrupted by transposon mutagenesis (*kfiB*, *kpsT*) and/or subject to gene knockout (*kfiB*,*C*) in this study are identified. HP—denote hypothetical proteins of unknown function **B)** Representation of main stages and associated K5 biosynthetic machinery (stages **1–3**). K5 assembly is localised to the cytoplasmic face of the inner membrane, and is underpinned by the formation of a biosynthetic complex which catalyses synthesis and export polysaccharide precursors for incorporation in the maturing capsule on the cell surface. During K5 assembly it is believed that a unified biosynthetic complex is developed which progressively catalyses main stages [1–3]. However, for clarity here we have separated each main stage of K5 synthesis and associated membrane complexes. **Stage 1)** Proteins encoded by *kpsF*,*U*,*C*,*S* are believed to be responsible for the initial generation of the phospatyidyl acceptor and Kdo linker (keto-3-deoxy-manno-2-octulosonic acid), upon which the polysaccharide chain is synthesised. **Stage 2)** Proteins encoded by *kfiA-D* are responsible for synthesis of the polysaccharide chain through addition of alternating units of GlcA (glucuronic acid) and GlcNAc (N-acetyl-glucosamine) from UDP-sugar precursors. **Stage 3)** Proteins generated by *kpsD*,*E*,*M*,*T* form an ABC transporter complex that translocates completed polysaccharide chains to the cell surface, in an energy dependant process.
(TIFF)

S1 Table Summary of mutant characteristics and experimental results. (PDF)

S2 Table Primers used in this study. (PDF)
