# Supplementary figures and images for: Disruption of Escherichia coli Nissle 1917 K5 Capsule Biosynthesis, through Loss of Distinct kfi genes, Modulates Interaction with Intestinal Epithelial Cells and Impact on Cell Health
Source: PLoS One. 2015 Mar 19;10(3):e0120430. doi: 10.1371/journal.pone.0120430 (PMC4366286; doi:10.1371/journal.pone.0120430)

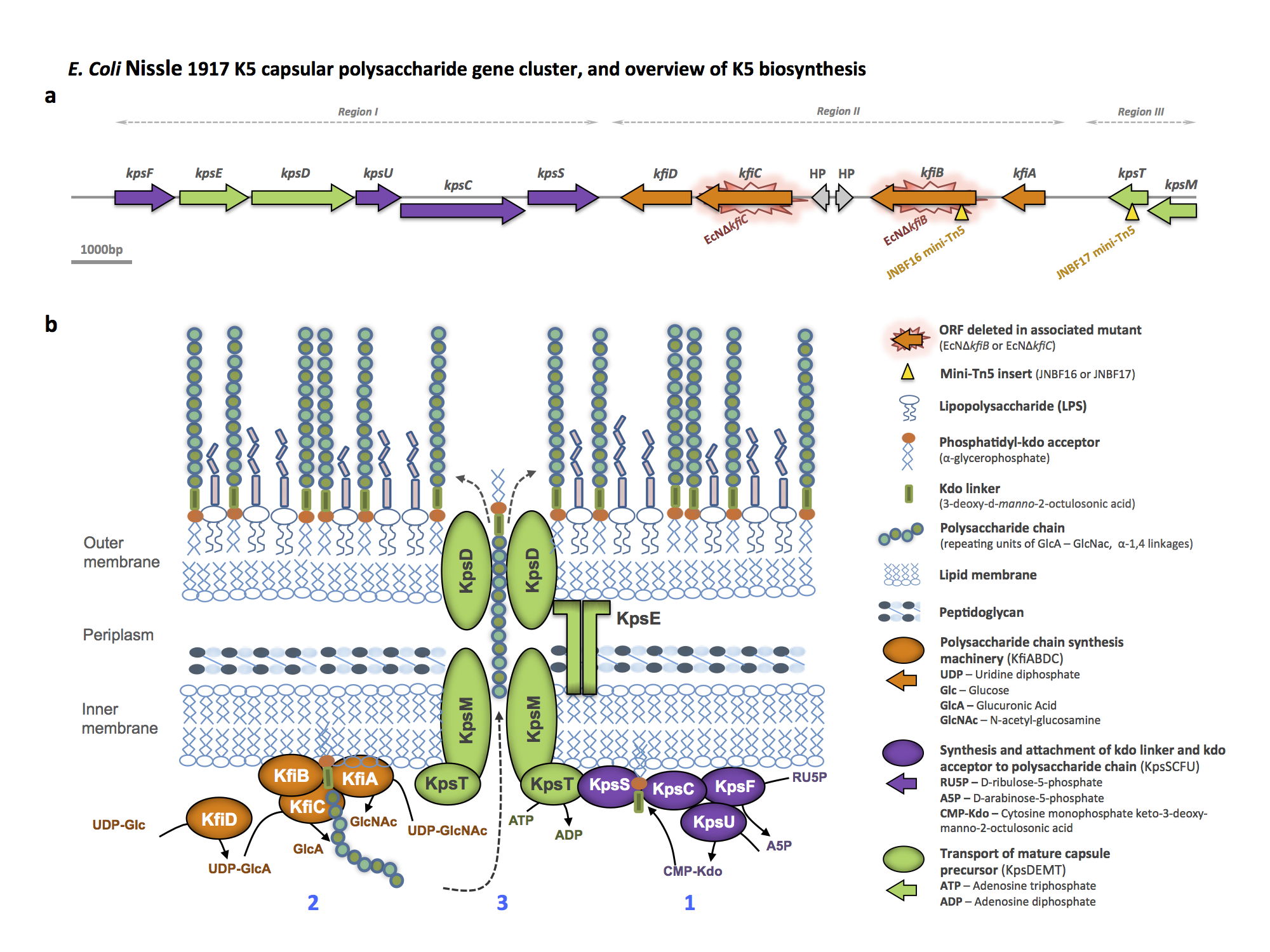

Supplement: S1 Fig — Diagrams show the genetic organisation of the K5 gene cluster in E. coli Nissle 1917 based on data from Cress et al. [28]; Grozdanov et al. [29], and an overview of the current model for K5 capsule biosynthesis and assembly adapted from Griffiths et al. [36]; Whitfield [41]; Petit et al. [42]; Bliss et al. [43]; Hodson et al. [44]; Corbett and Roberts [45]; Whitfield and Roberts [46]; Rigg et al. [47]; Whitfield and Willis [58]. A) Physical map of the EcN K5 capsular polysaccharide gene cluster. Region I (kpsF,E,D,U,C,S) and Region III (kpsM,T) encode elements of synthesis and export machinery, and are conserved among E. coli strains generating Group 2 polysaccharide capsules. Region II encodes K5 specific polysaccharide synthesis machinery (kfiA,B,C,D). Genes disrupted by transposon mutagenesis (kfiB, kpsT) and/or subject to gene knockout (kfiB,C) in this study are identified. HP—denote hypothetical proteins of unknown function B) Representation of main stages and associated K5 biosynthetic machinery (stages 1–3). K5 assembly is localised to the cytoplasmic face of the inner membrane, and is underpinned by the formation of a biosynthetic complex which catalyses synthesis and export polysaccharide precursors for incorporation in the maturing capsule on the cell surface. During K5 assembly it is believed that a unified biosynthetic complex is developed which progressively catalyses main stages [1–3]. However, for clarity here we have separated each main stage of K5 synthesis and associated membrane complexes. Stage 1) Proteins encoded by kpsF,U,C,S are believed to be responsible for the initial generation of the phospatyidyl acceptor and Kdo linker (keto-3-deoxy-manno-2-octulosonic acid), upon which the polysaccharide chain is synthesised. Stage 2) Proteins encoded by kfiA-D are responsible for synthesis of the polysaccharide chain through addition of alternating units of GlcA (glucuronic acid) and GlcNAc (N-acetyl-glucosamine) from UDP-sugar precursors. Stage 3) Pro [file pone.0120430.s001.tiff]
