# Supplementary material for: Disruption of Escherichia coli Nissle 1917 K5 Capsule Biosynthesis, through Loss of Distinct kfi genes, Modulates Interaction with Intestinal Epithelial Cells and Impact on Cell Health
Source: PLoS One. 2015 Mar 19;10(3):e0120430. doi: 10.1371/journal.pone.0120430 (PMC4366286; doi:10.1371/journal.pone.0120430)
Supplement: S1 Table — (PDF) [file pone.0120430.s002.pdf]

Supplementary Table 1: Summary of mutant characteristics and experimental results.

Assay/Figure	Wild-type	EcNΔ*kfiB*	EcNΔ*kfiC*	JNBF17 mini-Tn*5* KpsT
**Biofilm formation** (Table 1 & Fig. 2)	+	++	+	++
**ΦK5 sensitivity** (Fig. 2)	S	R	R	R
**Adherence to Caco-2** (Fig. 1 & 2)	+	++	+	+
**Apoptosis** (Fig. 3)	-	++	-/+	NT
**LDH release** (Fig. 3)	+	+++	++/-	NT
**COX-2 expression** (Fig. 3)	+	+	++	NT
**Caco-2 cell cytoskeleton and nuclear integrity** (Fig. 3)	Comparable to untreated cells	Increased incidence of condensed and/or fragmented nuclei. Considerable rearrangements in cell cytoskeleton	Occasional condensed/defragmented nuclei observed. Some indication of cytoskeletal alterations but largely comparable to wt treated cells	NT
**Cell morphology** (Fig. 4)	Predominantly short vegetative cells, but highly elongated cells commonly observed	Very short vegetative cells	Very short vegetative cells	Short vegetative cells with numerous elongated cells
**Aggregation** (Fig. 4)	No aggregation	Frequent large aggregates of closely associated cells common	Small aggregates of closely associated cells rarely observed	Frequent large aggregates of loosely associated cells, dominated by elongated morphologies.
**Motility (data not shown)**	+	-/+	+	+
**Predicted impact on K5 biosynthetic complex formation (KfiABCD complex)** (Fig. S1) *	Intact fully functional	Absence of KfiB. KfiA,D functional and localised to membrane. Unstable association of KfiC with KfiA, abolishing KfiC contribution to polymer synthesis	Absence of KfiC. KfiA, KfiB and KfiD functional but unable to localise to membrane resulting and form biosynthetic complex.	Intact fully functional

**Predicted impact on phosphatidyl acceptor and kdo linkage (KpsSCFU complex)** (Fig. S1) *****	Intact fully functional	Intact and functional, but polysaccharide chain synthesis not initiated/unstable	Intact and functional, but polysaccharide chain synthesis not initiated/unstable	Intact fully functional
**Predicted impact on Export machinery (KpsTMDE complex) (**Fig. S1**) ***	Intact fully functional	Intact fully functional	Intact fully functional	Channel formed but transport blocked by loss of KpsT.

* Predictions regarding impact of specific gene disruptions on the wider K5 biosynthetic machinery are based on previous studies examining loss of specific genes on the K5 assembly process, and draw upon studies by Petit *et al.* (24); Griffiths *et al.* (36); Bliss *et al.* (43); Hodson *et al.* (44); Rigg *et al*. (47); and comprehensive reviews by Whitfield (41); Corbett and Roberts (45); and Whitfield and Roberts (46).
