# Supplementary material for: Disruption of Escherichia coli Nissle 1917 K5 Capsule Biosynthesis, through Loss of Distinct kfi genes, Modulates Interaction with Intestinal Epithelial Cells and Impact on Cell Health
Source: PLoS One. 2015 Mar 19;10(3):e0120430. doi: 10.1371/journal.pone.0120430 (PMC4366286; doi:10.1371/journal.pone.0120430)
Supplement: S2 Table — (PDF) [file pone.0120430.s003.pdf]

Supplementary Table 2: Primers used in this study

Primer	Sequence (5'→3')	AT (°C)[a]	PCR product	Source
**NPT2_F1**<br>**NPT2_R1**	CTTGCTCGAGGCCGCGATTAAATT<br>TTCCATAGGATGGCAAGATCCTGG	62	Segment of mini-Tn*5* *nptII* gene.	Jones *et al.* (59)
**EcN*kfiB*_F**<br>**EcN*kfiB*_R**	TAGTCGGACATCCTGGCTCA<br>GCCCTTGATTTTAGCTCTCC	56.3	*kfiB* locus in EcN K5 biosynthesis cluster	This study
**EcN*kfiC*_F**<br>**EcN*kfiC*_R**	GGGACAAATATTGGTGCTCTTG<br>GTCTTGCCGCGATATCACTA	52.2	*kfiC* locus of EcN K5 biosynthesis cluster	This study
kfiB*.int_F** [1]<br>kfiB*.int _R** [1]	CAACAACAATTAACTTAAATGGAAGGGTAATGAATCCAAATATCGAATTA**AGTGTGCTGGAATTCGCCCT**<br>TCAGATAGTTGAACATTTTTTGAAAGAAATTGGCATGAACTCACCAAATT**CTGCAGAATTCGCCCTTCCT**	70.8	Construction of the *dif* $_{E.\ coli}$ *-cat-dif* $_{E.\ coli}$ cassette (from pTOPO-DifCAT plasmid) for deletion of a 1,552 -nt segment of *kfiB*.	This study
kfiC*.int_F** [1]<br>kfiC*.int _R** [1]	CTTCGAGATTGTGATATATATCCATCAGGTAGAGCTTCTGTTCCTTTATT**AGTGTGCTGGAATTCGCCCT**<br>CAATTCATAAGGAGAAAGTTGATCTTCAACATAAAAACTCGCCTTTAAAA**CTGCAGAATTCGCCCTTCCT**	70.8	Construction of the *dif* $_{E.\ coli}$ *-cat-dif* $_{E.\ coli}$ cassette (from pTOPO-DifCAT plasmid) for deletion of a 1, 257 -nt segment of *kfiC.*	This study
Xer-cise_F<br>***Xer-cise_R***	AGTGTGCTGGAATTCGCCCT<br>CTGCAGAATTCGCCCTTCCT	58.4	*dif* $_{E.\ coli}$ *-cat-dif* $_{E.\ coli}$ cassette control	This study
**RT*kfiA_F***<br>**RT*kfiA_R***	TGTTGGGATTCATGGCTGTA<br>TGCGATTGCTTGTGTTTCTT	52.2	Internal segment of *kfiA*	This study
**RT*kfiB_F***<br>**RT*kfiB_R***	GGCTGCATTATGGGAGGTAG<br>TGTTCCAACTGCTCTTGCAC	56.3	Internal segment of *kfiB*	This study
**RT*kfiC_F***<br>**RT*kfiC_R***	ATTGTCGCCCAAACAAAAAG<br>ACAATCATCGCACACGAGAA	52.2	Internal segment of *kfiC*	This study
**RT*kfiD_F***<br>**RT*kfiD_R***	TTGGTCTGAATACGCGTCAG<br>CCCGTTCCAAAGGTGAGTTA	56.3	Internal segment of *kfiD*	This study

Primer	Sequence (5'→3')	AT (°C)[a]	PCR product	Source
**PRL27Prmr 1**	GAGTCAGCAACACCTTCTTC	Manoil[2]	Mini-Tn*5* specific 1st round PCR	This study
**Primer 2a**	GGCCACGCGTCGACTAGTACNNNNNNNNNNAGAG		Degenerate 1st round primer	
**Primer 2b**	GGCCACGCGTCGACTAGTACNNNNNNNNNNACGCC		Degenerate 1st round primer	Manoil (55)
**Primer 2c**	GGCCACGCGTCGACTAGTACNNNNNNNNNNGATAT		Degenerate 1st round primer	
**PRL27Prmr 3**	AGCTTCAGGGTTGAGATGT		Mini-Tn*5* specific 2nd round PCR. Sequencing primer	
**Primer 4**	GGCCACGCGTCGACTAGTAC		2nd Round primer specific for 2a,b,c overhangs	

[a] AT - Annealing temperature applied in PCRs using listed primers
[1] For primers used for construction of deletion mutants (*kfiB*.int_F/R and *kfiC*.int_F/R; 70-nt in length), underlined sections showregions homologous to termini of target genes. Sections in bold show regions homolgous to the pTOPO-DifCAT vector.
[2] For primers used to amplify regions flanking mini-Tn*5* inserts, AT and PCR conditions are described in the original reference,Manoil (55).

59. Jones BV, Young R, Mahenthiralingam E, Stickler DJ. Ultrastructure of Proteus mirabilis swarmer cell rafts and role of swarming in catheter-associated urinary tract infection. Infect Immun. 2004; 72: 3941-3950.
